# The role of eating disorders features, psychopathology, and defense mechanisms in the comprehension of orthorexic tendencies

**DOI:** 10.1007/s40519-022-01417-3

**Published:** 2022-06-01

**Authors:** Giulia Costanzo, Daniela Marchetti, Giovanna Manna, Maria Cristina Verrocchio, Giorgio Falgares

**Affiliations:** 1 Department of Psychology, Educational Science and Human Movement, University of Palermo, Palermo, Italy; 2 Department of Psychological, Health and Territorial Sciences, G. d’Annunzio University of Chieti-Pescara, Via dei Vestini, 66100 Chieti, Italy

**Keywords:** Orthorexia, Eating disorders, Psychopathology, Defense mechanisms

## Abstract

**Purpose:**

Recent studies pointed out the importance to distinguish orthorexia nervosa (ON) from non-problematic forms of interest with healthy eating. This distinction needs to be further explored since it may favor a better comprehension of the relationship between orthorexic behaviors and psychopathology and lead to an improved understanding of the psychological processes implicated in ON. Therefore, the aim of the current study was to investigate the associations between ON and the core features of eating disorders (EDs), psychopathological symptoms and defense mechanisms, by differentiating three groups of individuals: an ON symptoms group, a healthy-eating control group, and a normal-eating control group.

**Methods:**

College students (*n* = 270, *M*_age_ = 21.57, SD = 2.16) were recruited from Palermo’s University, in the south of Italy, and were assigned to three groups: 52 in the ON symptoms group, 157 in the healthy-eating control group and 61 in the normal-eating control group. Participants completed four questionnaires assessing ON (EHQ-21), eating psychopathology (EDI-3), psychopathological symptoms (BSI) and defense mechanisms (DSQ-40).

**Results:**

Compared to the control groups, the ON symptoms group reported greater EDs’ features, higher psychopathological symptoms and greater employment of different neurotic and immature defense mechanisms. No differences were found between groups with regard to obsessive–compulsive symptoms.

**Conclusion:**

Our findings support the notion that ON behaviors should be carefully distinguished from non-problematic forms of interest with healthy eating. Indeed, results suggest that ON individuals are characterized by similar clinical features and defensive functioning as those observed in traditional EDs, indicating the importance of deepening our understanding of the relationship between these conditions.

**Level of evidence:**

Level V, descriptive cross-sectional study.

## Introduction

The term orthorexia nervosa (ON) was introduced by Steven Bratman [1] to describe a phenomenon characterized by an obsessional fixation on healthy and “clean” food and proper nutrition. It is a particular condition that, in certain situations, can become more pathological over time, leading to excessive concerns regarding the safety and quality of food preparations and to restrictive food-related behaviors [2]. That includes a rigid adherence to inflexible self-imposed dietary rules, which can lead to impairments in important areas of functioning and to clinically significative consequences, including malnutrition and severe weight loss, medical complications, great intrapersonal and emotional distress (i.e., anxiety, depression, shame, feelings of guilt, sense of personal impurity when healthy eating routines are transgressed) and social isolation [3, 4]. Despite all these aspects being widely acknowledged, ON has not been yet recognized as a mental disorder by official nosographic classifications, although some diagnostic criteria have been recently proposed [3].

Recent studies highlighted the necessity to differentiate this pathological obsession with healthy eating from other non-problematic eating behaviors, which instead are exclusively aimed at the promotion of one’s general health and well-being [5, 6]. This is in accordance with Barrada and Roncero’s 2018 work [7], in which the authors tried to expand the conceptualization of the orthorexic condition by distinguishing two forms of orthorexia: ON, which represents a pathological and overwhelming preoccupation with healthy diet as previously described, and healthy orthorexia (HeOr), which indicates a healthy interest in proper nutrition.

This distinction may be helpful for three different reasons. First, it may lead to greater accuracy in the diagnostic evaluation of this particular condition [7]. Second, it may favor a better comprehension of both the differences and the overlapping aspects between problematic forms of orthorexia (ON) and other psychopathological entities (above all, eating disorders and obsessive–compulsive disorder) [8–10]. Third, it may lead to an improved understanding of the psychological processes implicated in the onset and in the maintenance of ON, which need further exploration [11].

### Orthorexia and eating disorders

While non-problematic forms of interest in healthy eating have been clearly differentiated from eating disorders (EDs) [7, 12], the nature of the relationship between EDs and ON remains controversial, to the extent that it is still unclear whether ON should be considered or not as an epiphenomenon or as a prodromic condition of a specific ED [13]. In effect, ON shares many similarities with EDs, such as a lack of pleasure related to food consumption and the need to control food intake as a strategy to reach a sense of control over one's own life and to improve self-esteem and self-realization [10, 14]. This similarity is supported by several studies, which highlight how ON and traditional EDs (particularly, anorexia nervosa/AN) seem to share some important features such as perfectionism, appearance orientation, drive for thinness, interpersonal distrust, interoceptive awareness and difficulties in emotion regulation [11, 15–17].

Nevertheless, these conditions also present some differences. For example, orthorexic behaviors are believed to be driven by the goal of achieving the ideal of a pure and healthy body, rather than by the need to lose weight in order to reach a distorted ideal in terms of body image [18]. Furthermore, according to McComb and Mills (10), while individuals with EDs (and particularly with AN) are motivated to hide their pathological eating behaviors, orthorexic individuals tend to flaunt their eating habits, showing feelings of moral superiority. Finally, while EDs are notoriously characterized by body image disturbances, research has provided mixed results with regard to ON [8, 17, 19]. These controversial results may also be due to the use of diagnostic tools, particularly the ORTO-15, whose validity and reliability have been frequently questioned, as well as their ability to distinguish between ON behaviors and non-pathological healthy eating [20]. We believe that this represents a limitation of previous studies that needs to be addressed by further exploring the associations between ON and EDs through different measures.

### Orthorexia and psychopathology

Some researchers have considered ON as a subtype of obsessive–compulsive disorder (OCD) in light of the several obsessive–compulsive features that seem to characterize orthorexic individuals, such as intrusive thoughts about food and health, exaggerated preoccupations over contamination and impurity and ritualized patterns of food preparation, along with a prominent tendency to spend extra time in cataloging, weighing and measuring food and in planning future meals [3, 13, 21]. Nevertheless, in order to consider a food-related problematic condition as a subtype of OCD, its obsessive–compulsive symptoms must not be uniquely related to food, since exaggerated and ritualistic focus on eating and on the quantity and quality of meal preparation represents a core feature of EDs [9]. Thus, while some studies [10, 22] found that individuals with ON were more likely to report higher OCD symptoms, food-related or not, other studies [9, 23] observed that the association between ON and OCD became smaller or absent when controlling for ED symptoms. This suggests that the presence of obsessive–compulsive tendencies in individuals with ON may be due to the overlap between ON and EDs and, consequently, to the high rates of comorbidity between EDs and OCD [24, 25]. Therefore, we believe that more information is needed to deepen our understanding of this link, also in light of the above-mentioned limitations of previous studies that used the ORTO-15 [20].

Besides the association with OCD, it seems that the problematic forms of orthorexia are also related to higher levels of general psychopathological severity [26]. For example, some studies have highlighted a close association between ON and depressive symptoms [27, 28], to the extent that Strahler et al. [13] found that 48% of orthorexic participants in their study suffered from at least moderate depression. Other studies have highlighted the presence in ON individuals of both trait anxiety [13] and anxiety symptoms referred to specific domains, including health preoccupation [29], appearance anxiety [30] and social physique anxiety [31]. Moreover, other studies have underlined the possible role of personal distress in the context of somatization, finding that ON correlated with more somatic symptoms not attributable to physiologic abnormalities [32]. It is, therefore, possible that individuals with somatic symptoms try to cope with their diseases by modifying their eating behaviors and by adopting a rigid healthy diet [33]. Finally, Koven and Abry [21] suggested that ON may also represent a prodrome of more severe psychopathology (i.e., psychotic conditions), as observed by Saddicha et al. [34] in a case study of a woman whose orthorexic behaviors constituted a prodromic phase of schizophrenia. In light of these findings, it would be of interest to further investigate the presence of these conditions among ON individuals.

### Psychological processes implicated in ON

While most of the literature has mainly focused on the comorbidity between ON and other psychopathological entities, the psychological processes underlying the mental functioning of ON individuals have been less explored. According to Lingiardi and McWilliams [35], mental functioning is the result of the integration of aspects such as, for example, mentalization, emotion and impulse regulation, defensive functioning, capacity for intimacy and self-observing abilities. To the best of our knowledge, among all these processes, only emotion regulation has been investigated in relation to ON [11]. Instead, we believe that exploring the defense mechanisms involved in this condition may offer crucial information for the comprehension of the psychological functioning of these individuals, with important implications for etiology, assessment and intervention [36, 37]. Indeed, the evaluation of defense mechanisms may be helpful to inform intervention strategies aimed at helping ON individuals to make sense of their experience, symptoms and behaviors, and therefore, to deal with emotionally difficult situations in more adaptive ways [38]. Moreover, the evaluation of this aspect of the orthorexic mental functioning may allow to compare it to what previous studies have found about AN, BN and OCD psychological processes. We believe that this would make less controversial the nature of the relationship between ON and these psychopathological conditions.

Defense mechanisms are automatic and unconscious psychological processes that affect the individual adjustment to internal or external stressors or to emotional conflicts [39]. They are involuntary strategies that function to alter the way these stressors and conflicts are perceived, with the aim to reduce excessive anxiety and maintain one’s self-esteem and the integration of Self [40, 41]. Vaillant [42] proposed a hierarchical classification of defense mechanisms based on their maturity levels, distinguishing psychotic, immature, neurotic and mature defense styles. The choice of a particular defense style significantly contributes to individual differences in personal responses to stressful events and to intrapsychic or interpersonal conflicts, with the possibility to intercept healthy and pathological styles [40]. In effect, while mature defense mechanisms are positively associated with better levels of psychosocial adjustment and personal satisfaction [43], neurotic, immature and psychotic defense mechanisms seem to be involved in both the onset and in the maintenance of psychopathology [41].

Although defense mechanisms have not been explored in relation to ON, hypotheses may be formulated on the basis of previous research on people with EDs and OCD, which have been discussed as two overlapping domains with ON. These studies found that individuals with EDs and OCD are characterized by the prominent use of immature and neurotic defense mechanisms, such as denial, projection, passive aggression, undoing, somatization, splitting and displacement for EDs [36, 44], and acting out, reaction formation, undoing, rationalization and denial for OCD [45, 46]. Although the explorative nature of the study does not consent to formulate accurate hypotheses on the relationship between ON and specific defense mechanisms and styles, we would expect to find in ON individuals similar defenses to those previously mentioned in relation to EDs and OCD (i.e., denial, passive aggression, undoing, rationalization, somatization, displacement, and acting out), as well as more neurotic and immature defense styles.

Please refer to Gabbard’s classification of defense mechanisms for a more exhaustive discussion [47].

### The present study

The aim of the current study was to explore how the core features of EDs, psychopathological symptoms and defense mechanisms are associated with ON. To this purpose, we have replicated the research design proposed in a study by Oberle et al. [18], comparing the ON symptoms group to two different control groups: a healthy-eating control group and a normal-eating control group. The healthy-eating control group was composed of individuals interested in healthy eating who do not experience negative consequences as a result of their eating habits, in line with literature’s suggestions regarding the necessity to differentiate ON from non-problematic forms of interest in healthy eating [5]. The normal-eating control group, instead, was composed of individuals not interested in healthy eating, who scored lowest on a measure of ON symptomatology.

With regard to the characteristics of eating psychopathology, based on several studies showing an overlap between EDs and ON (for a review, see ref. [10]), we hypothesized that the ON symptoms group, in comparison to both control groups, would report more ED core features, specifically the drive for thinness and bulimia, while we expected no differences in body dissatisfaction. Indeed, in light of inconsistent results in the literature [8, 17], we believe that the absence of concerns about body image could allow us to differentiate ON from other EDs, particularly AN. Moreover, a higher eating disorder risk was expected in the ON symptoms group in comparison to both control groups.

Regarding general psychopathological symptoms, we hypothesized that the ON symptoms group would not report significantly higher levels of OCD symptoms compared to both control groups, in line with literature results suggesting that the presence of obsessive–compulsive characteristics in ON individuals may be exclusively due to the high rates of comorbidity with other EDs [9, 23]. Moreover, we hypothesized that the ON symptoms group would report more symptoms of depression, anxiety, somatization and psychoticism in comparison to both control groups, especially the healthy-eating control group.

Finally, regarding defense mechanisms, based on research showing that other EDs are associated with neurotic and immature defense mechanisms [36], we hypothesized that the ON symptoms group would report more neurotic and immature defense mechanisms in comparison to both control groups.

## Methods

### Participants

Participants were recruited from a variety of undergraduate courses (i.e., humanities, scientific, social, and health) at Palermo’s University*,* a large university in the southern region of Italy. Inclusion criteria were:  ≥ 18 years; capability to read and understand Italian; and signed informed consent. Based on criteria described below, from the 440 participants who completed the survey, data were retained for 270 participants, 52 who comprised the ON symptoms group, 157 who comprised the healthy-eating control group and 61 who comprised the normal-eating control group. This sample of 270 students included 70 men and 200 women, whose ages ranged from 18 to 37 years (*M* = 21.57, SD = 2.16). Three criteria were used for the assignment of participants into the ON symptoms group [18], as follows: (1) the total score had to be within the top 25th percentile on a valid and reliable measure of ON symptomatology, the Eating Habits Questionnaire-21 (EHQ-21) [48]; (2) the average Likert score for the EHQ-21 Knowledge scale had to be at least 2, indicating that the items about healthy-eating knowledge and behaviors did describe them at least slightly; (3) the average Likert score for the EHQ-21 Problems scale had to be at least 2, reflecting that the items about problems resulting from their healthy-eating behaviors did describe them at least slightly. Three criteria were used for the selection and allocation of participants into the normal-eating control group [18], as follows: (1) the total EHQ-21 score had to be within the bottom 25th percentile; (2) the average Likert score for the EHQ-21 Knowledge scale had to be less than 1.5, indicating that the items about healthy-eating knowledge and behaviors did not describe them sufficiently well; (3) the average Likert score for the EHQ-21 Problems scale also had to be less than 1.5, reflecting that the items about problems due to healthy-eating behaviors did not describe them sufficiently well. Finally, two criteria were used for the assignment of participants into the healthy-eating control group [18], as follows: (1) the average Likert score for the EHQ-21 Knowledge scale had to be at least 2 indicating that the items about knowledge and behaviors did describe them at least slightly; (2) the average Likert score for the EHQ Problems scale had to be less than 1.5, showing that the items about problems due to healthy-eating behaviors did not describe them sufficiently well. Participants who did not meet criteria for any of the three groups (*N* = 170) were excluded.

### Measures

#### Socio-demographics

Respondents were asked to indicate their gender, age, and income level. Respondents were also asked to indicate if they were following a diet and if they regularly practiced physical activity.

#### Eating Habits Questionnaire-21

Orthorexia was assessed using the Italian version of the 21-item Eating Habits Questionnaire (EHQ-21) [48, 49]. This self-report measure explores (a) beliefs related to healthy eating (via the “Knowledge” subscale; example item: “*My eating habits are superior to others*”), (b) feelings associated with healthy eating (via the “Feelings” subscale; example item: “*I feel in control when I eat healthily*”), and (c) problems related to these behaviors (via the “Problems” subscale; example item: “*I turn down social offers that involve eating unhealthy food*”). According to Zickgraf et al. [50], specifically the “Problems” subscale is particularly valuable in discriminating the orthorexic condition from normal healthy-eating behaviors. All items are rated on a four-point Likert scale, ranging from 1 (*false, not at all true*) to 4 (*very true*). The original version of EHQ-21 has good internal consistency and test–retest reliability, as well as adequate convergent, discriminant and criterion-related validity [48]. In the present sample, Cronbach’s alpha values were 0.80, 0.63, and 0.83 for the Knowledge, Feelings, and Problems subscales, respectively.

Based on the EHQ-21 scores, participants were grouped into the ON symptoms, normal-eating control and healthy-eating control groups by applying criteria established by Oberle and colleagues [18].

#### Eating Disorder Inventory-3

Symptoms and psychological features of EDs were assessed using the Italian adaptation of the third version of the Eating Disorder Inventory (EDI-3) [51, 52], which is a self-report questionnaire widely used both in clinical and non-clinical settings. It is composed of 91 items divided into 12 subscales, consisting of three eating-disorder-specific scales (drive for thinness, bulimia and body dissatisfaction; the combination of these three subscales produces the eating disorder risk composite score, which provides a global measure of ED risk) and nine general psychological scales (low self-esteem, personal alienation, interpersonal insecurity, interpersonal alienation, interoceptive deficits, maturity fears, perfectionism, asceticism and emotional dysregulation) that, although not specific, are relevant in the development and maintenance of EDs. All items were rated on a six-point Likert scale, ranging from 1 (*never*) to 6 (*always*). For the purpose of our study, we used the three eating-disorder-specific scales and the eating disorder risk composite score. In the current sample, Cronbach’s alpha values were 0.91, 0.82, and 0.86 for the drive for thinness, bulimia and body dissatisfaction scales, respectively. Cronbach’s alpha for the eating disorder risk composite score was 0.93.

#### Brief Symptom Inventory

The Italian version of the Brief Symptom Inventory (BSI) [53, 54] was used to assess general psychopathology. It is a 53-item self-report inventory covering nine symptom dimensions: somatization, obsession-compulsion, interpersonal sensitivity, depression, anxiety, hostility, phobic anxiety, paranoid ideation and psychoticism. The BSI also includes three global indices of distress: (a) Global Severity Index (GSI), which measures current or past level of symptomatology; (b) Positive Symptom Distress Index (PSDI), which assesses the intensity of symptoms; and (c) Positive Symptom Total (PST), which measures the number of reported symptoms. Each item is rated on a five-point Likert scale, ranging from 0 (*not at all*) to 4 (*extremely*). The BSI is the short form of the SCL-90-R [55], which assesses the same dimensions. The scales show acceptable levels of validity and reliability, as seen in previous studies on the Italian general population and medically ill patients [56, 57]. In the present sample, Cronbach’s alpha values for the nine symptoms scales ranged between 0.62 and 0.84. Cronbach’s alpha for the GSI was 0.95.

#### Defense Style Questionnaire-40

The Italian adaptation of the Defense Style Questionnaire (DSQ-40) [58, 59] was used to investigate individual defensive functioning by measuring the conscious derivatives of different defense mechanisms. It is a self-report questionnaire composed of 40 items which assess 20 defenses (two items for each one), which in turn are categorized into 3 different defense styles: (a) mature, which includes sublimation, humor, anticipation and suppression; (b) neurotic, which includes undoing, pseudo-altruism, idealization and reaction formation; and (c) immature, which includes projection, passive aggression, acting out, isolation, devaluation, autistic fantasy, denial, displacement, dissociation, splitting, rationalization and somatization. Each item is rated on a nine-point Likert scale, ranging from 1 (*strongly disagree*) to 9 (*strongly agree*). Final scores for each individual defense mechanism were derived by calculating the average of the two relevant items for each defense, while the final scores for each defense style were derived by the average of the individual defense scores contributing to that factor. Higher scores indicate a greater usage of the target defense mechanism or style. In the present sample, Cronbach’s alpha values were 0.55, 0.52, and 0.80 for the mature, neurotic, and immature defense styles, respectively.

### Procedure

The Ethical Committee of the Department of Psychological, Health and Territorial Sciences at G. d’Annunzio University of Chieti-Pescara (protocol number 21010) approved the study and all procedures were performed in accordance with the ethical principles for psychological research, following the Declaration of Helsinki and its revisions [60] as well as the ethics guidelines of the American Psychological Association (APA) [61].

Data were collected during class time where participants were informed about the purpose of the research, the voluntary nature of participation, the anonymity of responses, the option to withdraw at any time, and finally were asked to participate in the study. More than 95% agreed to participate and written informed consent was obtained from all of them prior to collecting data in groups of 30–35 students in a large space to ensure anonymity. All students were invited to call the research lab for any further information about the study. Participants did not receive any form of compensation for their participation.

### Statistical analysis

The study used a between-subject design with group as the independent variable and DSQ defenses and defense style scores, BSI symptoms and GSI scores, EDI-3 drive for thinness, bulimia and body dissatisfaction, and eating disorder risk composite scores as the dependent variables. Analyses were conducted with IBM SPSS Statistics 26 for Windows. Descriptive data, means (M), standard deviations (SD) and frequencies are reported. Non-parametric Kruskal–Wallis tests with factor group for independent samples were used due to large differences in sample sizes with a *p* value of 0.05. As a post hoc test, pairwise comparisons with Bonferroni’s adjusted alpha of 0.017 were computed to determine differences between the groups.

## Results

First, we compared ON symptoms, normal-eating control, and healthy-eating control groups on socio-demographics. The three groups were equivalent for gender [*χ*^2^(2) = 0.48, *p* = 0.787], age [*χ*^2^(2) = 0.21, *p* = 0.901] and income [*χ*^2^(2) = 3.85, *p* = 0.427]. Significant differences characterized the three groups in terms of diet [*χ*^2^(2) = 59.53, *p* = 0.000] and physical activity [*χ*^2^(2) = 12.15, *p* = 0.002], with higher frequencies of both habits in the ON symptoms group compared to both control groups (see Table 1 for further data).

**Table 1:** Socio-demographics of the study groups

	ON symptoms group	Healthy-eating control group	Normal-eating control group
	*n* = 52 % or M (SD)	*n* = 157 % or M (SD)	*n* = 61 % or M (SD)
Gender			
Female	75	72.6	77
Male	25	27.4	23
Age	21.29 (1.47)	21.69 (2.53)	21.51 (1.57)
Income			
Low	26.5	35.7	43.9
Medium	44.9	42.9	36.8
High	28.6	21.4	19.3
Diet^a^			
Yes	55.8	14	1.6
No	44.2	86	98.4
Physical activity			
Yes	59.6	47.8	27.9
No	40.4	52.2	72.1

^a^Low-carbohydrate, low-fat, high-protein, vegetarian, vegan, or Mediterranean diet

Second, we examined group differences in eating and general psychopathology. Table 2 shows the descriptive data and Kruskal–Wallis test results for the main effects. Regarding post hoc analyses in EDI-3 scores, the ON symptoms participants were higher in drive for thinness, body dissatisfaction and the eating disorder risk composite than the control groups, while higher scores on bulimia were found compared to healthy-eating controls only. No differences between control groups emerged in symptoms of EDs. Looking at the post hoc results for BSI scores, the ON symptoms group scored significantly higher in somatization, anxiety, paranoid ideation, psychoticism and GSI compared to the healthy-eating controls, while the normal-eating controls were in between the other two groups in these scores. Furthermore, the ON symptoms group had higher scores than the normal-eating controls in phobic anxiety, while they did not differ from the healthy-eating group. No differences were found between groups in obsession-compulsion, interpersonal sensitivity, depression and hostility.

**Table 2:** Group differences in eating disorder features and psychopathological symptoms

	ON symptoms group (ON) *n* = 52		Healthy-eating control group (HE) *n* = 157		Normal-eating control group (NE) *n* = 61		Kruskal–Wallis test		Post hoc differences
	*M*	(SD)	*M*	(SD)	*M*	(SD)	*χ*^2^ (2)	*p*	
BSI									
Somatization	0.72	0.68	0.46	0.52	0.55	0.64	7.136	0.028	ON > HE
Obsession–compulsion	1.33	0.87	1.12	0.78	1.18	0.76	2.266	0.322	–
Interpersonal sensitivity	0.97	0.82	0.81	0.72	0.89	0.78	1.412	0.494	–
Depression	1.08	0.84	0.87	0.75	0.94	0.78	2.348	0.309	–
Anxiety	1.24	0.93	0.85	0.63	0.97	0.68	6.828	0.033	ON > HE
Hostility	0.89	0.75	0.66	0.65	0.74	0.57	4.980	0.083	–
Phobic anxiety	0.48	0.58	0.31	0.48	0.23	0.38	7.296	0.026	ON > NE
Paranoid ideation	1.19	0.82	0.87	0.71	0.90	0.74	6.697	0.035	ON > HE
Psychoticism	0.87	0.75	0.60	0.63	0.70	0.65	5.383	0.041	ON > HE
Global severity index	0.98	0.61	0.73	0.51	0.79	0.52	6.992	0.030	ON > HE
EDI-3									
Eating disorder risk composite	84.10	25.00	60.69	19.25	61.89	22.42	33.680	0.000	ON > HE, NE
Drive for thinness	28.96	9.05	17.85	7.81	16.69	7.92	52.868	0.000	ON > HE, NE
Bulimia	18.37	8.29	13.97	4.68	16.13	6.21	14.333	0.001	ON > HE
Body dissatisfaction	36.77	11.52	28.87	10.33	29.07	10.83	19.939	0.000	ON > HE, NE

*BSI* Brief Symptom Inventory, *EDI-3* Eating Disorder Inventory-3

Finally, we examined group differences on single defenses and related mature, neurotic and immature defense styles (see Table 3). Regarding mature defenses, the post hoc analyses on the anticipation and suppression scores indicated that the ON symptoms participants reported significantly higher scores on anticipation than normal-eating controls, but they did not differ from controls in suppression scores. The healthy-eating controls reported significantly higher scores on the use of anticipation and suppression compared to the normal-eating controls. Considering the mature defense style score, the healthy-eating control group had significantly higher scores than the normal-eating group. No differences in sublimation and humor scores were found between groups.

**Table 3:** Group differences in defense scales and styles

	ON symptoms group (ON) *n* = 52		Healthy-eating control group (HE) *n* = 157		Normal-eating control group (NE) *n* = 61		Kruskal–Wallis test		Post hoc differences
	*M*	(SD)	*M*	(SD)	*M*	(SD)	*χ*^2^ (2)	*p*	
DSQ-40									
Mature defense style	4.89	1.02	4.95	1.04	4.35	1.12	11.998	0.002	HE > NE
Sublimation	3.85	1.88	3.92	1.99	3.30	1.63	4.300	0.117	–
Humor	5.89	1.82	6.03	1.64	5.66	2.08	1.225	0.542	–
Anticipation	5.60	1.58	5.45	1.46	4.80	1.73	9.225	0.010	ON > NE; HE > NE
Suppression	4.24	1.60	4.43	1.64	3.63	1.67	9.887	0.007	HE > NE
Neurotic defense style	4.37	1.28	4.20	1.13	3.76	1.17	7.202	0.027	ON > NE
Undoing	4.29	1.73	4.01	1.68	3.50	1.87	6.197	0.031	ON > NE
Pseudo-altruism	4.33	1.60	3.90	1.80	3.53	1.62	5.627	0.060	–
Idealization	4.28	2.01	4.20	2.02	3.53	1.93	5.869	0.053	–
Reaction formation	4.60	1.73	4.71	1.80	4.47	1.95	0.635	0.728	–
Immature defense style	3.83	0.93	3.52	0.93	3.42	1.08	6.764	0.034	–
Projection	3.00	1.59	2.81	1.59	2.80	1.64	0.763	0.683	–
Passive aggression	3.97	1.77	3.40	1.83	3.89	2.00	6.167	0.046	ON > HE
Acting out	4.37	1–82	3.91	1.81	4.42	1.71	4.757	0.093	–
Isolation	4.30	2–06	3.94	2.21	3.84	2.16	1.752	0.416	–
Devaluation	4.24	1.66	4.36	1.61	3.54	1.64	10.477	0.005	HE > NE
Autistic fantasy	4.02	2.18	3.61	2.34	3.66	2.29	1.755	0.416	–
Denial	2.76	1.43	2.43	1.46	2.21	1.51	5.408	0.067	–
Displacement	3.14	1.67	2.67	1.53	2.61	1.82	4.798	0.091	–
Dissociation	3.34	1.53	3.19	1.73	2.67	1.55	6.658	0.036	ON > NE
Splitting	3.98	2.00	3.55	2.05	3.29	2.29	4.389	0.111	–
Rationalization	4.62	1.67	5.05	1.76	4.69	1.84	2.965	0.227	–
Somatization	4.21	2.00	3.32	1.83	3.40	2.16	8.613	0.013	ON > HE, NE

*DSQ-40* Defense Style Questionnaire-40

Comparing groups in terms of neurotic defenses, the post hoc analyses showed that the ON symptoms groups had higher scores in the undoing defense and the neurotic defense style compared to the normal-eating control group, while no differences emerged compared to the healthy-eating control group. The three groups were also comparable in their scores for pseudo-altruism, idealization and reaction formation defenses.

Regarding immature defenses, the ON symptoms participants scored significantly higher in somatization compared to both control groups, while higher scores for passive aggression emerged in comparison with the healthy-eating controls and higher scores in dissociation versus the normal-eating controls. Furthermore, the healthy-eating group scored significantly higher in devaluation defense than the normal-eating group. No other difference emerged between groups in the immature defenses and style scores.

## Discussion

This study explored the association of ED features, psychopathological symptoms and defense mechanisms with orthorexic tendencies by comparing three different groups: an ON symptoms group, a healthy-eating control group and a normal-eating control group.

With regard to ED features, we hypothesized that the ON symptoms group would report more drive for thinness and bulimia compared to both control groups, while we expected no differences in body dissatisfaction. Our results partially supported the hypotheses by showing higher levels of drive for thinness and overall ED risk, but also body dissatisfaction, in ON participants with respect to both control groups. Bulimia symptoms, instead, were higher in the ON group only compared to healthy-eating participants. No differences in ED features emerged between the healthy and normal-eating control groups. These findings converge with prior studies underlining the high prevalence in ON individuals of concerns about weight loss and body image, as typically studied in AN individuals [10, 17], as well as the presence of an increased ED risk. Moreover, given that the association between bulimia symptoms and emotion dysregulation is empirically supported [62], the presence of bulimia symptoms in ON participants is not surprising in light of the emotion regulation deficits that seem to characterize these individuals [11].

Taken together, this first set of results seems to support a representation of ON as a condition hard to differentiate from other EDs. Specifically, drive for thinness and body dissatisfaction in ON may suggest that the dietary behaviors of these individuals are only apparently unrelated to weight concerns. Indeed, excessive focus on healthy foods may act as a means to disguise thinness-related motivations, which are thus expressed in a socially acceptable way. This may be due to the awareness of the dangers of EDs in western culture, which may make the drive for thinness—apparently in opposition to the “drive for health”—an unacceptable motivation for restrictive dietary behaviors [63]. However, in practice, these two motivations (drive for thinness and drive for health) may be strictly related, and thus hard to discriminate, given that in our society health is usually equated with thinness, making more controversial the possibility to distinguish between ON and AN.

As hypothesized, the results regarding psychopathological symptoms did not show significant differences between groups with regard to OCD symptoms. These findings seem to support the notion that the obsessive–compulsive characteristics typically found in ON may be uniquely related to food concerns (e.g., ritualized patterns of food preparation, exaggerated focus on eating) and not to full-blown OCD, exclusively due to the presence of ED core features in ON individuals. Moreover, our results underlined significantly higher levels of anxiety, somatization, paranoid ideation and psychoticism in ON individuals compared to healthy-eating controls. Taken together, these findings indicate higher psychopathological severity in ON, as also emerged in the GSI score, which suggests that orthorexic behaviors may serve as dysfunctional coping strategies to manage emotional distress. Conversely, lower levels of psychopathological severity in the healthy-eating condition may indicate that more flexibility related to healthy-eating behaviors may serve as a protective factor against emotional distress, thereby enhancing psychological well-being. It is, therefore, possible that the differences between these groups with regard to psychopathology are attributable to underlying differences in terms of psychological functioning, which may be characterized by a greater rigidity in ON individuals.

With regard to defense mechanisms, we hypothesized that the ON symptoms group would report more neurotic and immature defense mechanisms compared to both control groups. Our results partially supported these hypotheses. Specifically, the ON symptoms group seems to be more characterized by a neurotic defense style only compared to normal-eating controls. Moreover, no significant differences were found between groups with regard to the immature defense style, although our results seem to indicate that ON individuals show a greater tendency to recur to immature mechanisms in comparison to both control groups. These results allowed us to obtain more information about orthorexic mental functioning, suggesting that the behavioral inflexibility that characterizes these individuals may be attributed to a more general alteration of reality testing due to the use of less mature defense mechanisms [64, 65]. However, the explorative nature of our suggestions regarding orthorexic defense mechanisms should be underlined, given that no previous studies have investigated them.

Considering specific defense mechanisms, the results highlight the greater use of somatization by the ON symptoms group compared to both control groups. Specifically, somatization can be defined as the tendency to express psychological distress and painful emotions through physical and organic symptoms [47]. It is, therefore, possible that, in orthorexic individuals, negative affects are transformed into painful physical states through the mechanism of somatization, making rigid healthy eating a means to mitigate physical distress. Alternatively, it is possible that orthorexic eating pathology represents by itself a somatic response to emotional distress, which in turn may be due to an unresolved intrapsychic conflict. This latter interpretation may bring orthorexic psychological functioning closer to that observed in anorexic pathology. Indeed, among maladaptive defense mechanisms, Coveney and Olver [36] have highlighted that somatization was the most significant defense observed in individuals with AN traits. This would make the boundaries between these two conditions increasingly less clear-cut.

Considering the comparison between the ON symptoms group and normal-eating controls, our findings show a significantly greater employment of anticipation, undoing and dissociation mechanisms by ON subjects. Specifically, with regard to anticipation, it describes the tendency to perceive future danger both affectively and cognitively, in order to be emotionally prepared for something that might happen [41]. It is, therefore, possible to suggest that negative thoughts about future health conditions may lead orthorexic individuals to recur to rigid healthy-eating habits to prevent possible physical diseases. Undoing, instead, refers to the tendency to engage in behaviors with the symbolic aim to revert or remove disturbing thoughts or actions previously experienced and/or performed [47]. It may occur when orthorexic individuals employ self-punishing behaviors (i.e., stricter diets, cleansing fasts, etc.) following dietary violations [21]. This defense mechanism may be similar to the one observed in anorexic or bulimic individuals that apply compensatory behaviors, such as vomiting and excessive exercise, when eating restrictions are transgressed [66]. Accordingly, previous studies [44, 67] have found that individuals with EDs reported higher levels of undoing, which would serve as a way to minimize threatening affects related to food. With regard to dissociation, it refers to a temporary alteration of the normal integration of thoughts, feelings and experiences into the stream of consciousness, which has the aim to protect the person from unpleasant emotions and memories [68]. It is, therefore, possible to suggest that orthorexic restrictive eating behaviors may serve as a means to dissociate disturbing emotions and restore a sense of control.

Finally, considering the comparison between the ON symptoms group and the healthy-eating controls, our findings show significant employment of passive aggression by ON subjects. Passive aggression consists of the inability to openly express feelings of anger, which are thus expressed indirectly. This defense seems to further bring our study’s orthorexic subjects closer to anorexic individuals, as suggested by past research [36, 44] that found higher levels of passive aggression associated with AN traits. Indeed, AN is characterized by the presence of internalized and unexpressed feelings of anger and resentment, which may be due to family pressures aimed at denying the individual’s autonomy and needs. For this reason, anorexic symptomatology may be considered as an act of indirect aggression towards the family [69]. Similarly, orthorexic behavior may represent a means for the individual to indirectly express his/her own aggression and self-definition need in the family context.

### Strength and limits

A number of study limitations need to be addressed. First, the cross-sectional nature of this research does not allow us to examine causal relationships among study variables. Future research would benefit from a longitudinal design for a better assessment of causality. Second, the study’s generalizability may be limited by the sample of university students in the southern region of Italy. Indeed, different results could be obtained with participants from different geographical regions and cultural and educational backgrounds. Moreover, the distribution of our participants by gender was unbalanced, with a prevalence of female participants. This is a further issue that could bias results and limit generalizability. Future research would benefit from the replication of this study with more heterogeneous and representative groups of people. Third, we only administered self-report questionnaires, which may be sensitive to social desirability bias, possibility inflating some of the associations among variables. Future research should use a multiple method approach, including qualitative interviews. Finally, we used the EHQ to differentiate the participants into groups, but this may represent a limitation given that the EHQ was not intended to assess non-pathological forms of interest in healthy eating. The Teruel Orthorexia Scale (TOS) [7] would have been the gold standard for this purpose, but it does not still exist an Italian version of this measure. Future research may thus consider replicating this study using the TOS.

This is, to our knowledge, the first study introducing defense mechanisms to explore orthorexic mental functioning, whose comprehension may have important implications for etiology, assessment and intervention. Particularly, it may be useful in clinical settings to help individuals with orthorexic tendencies to better understand feelings and intentions that underlie their dysfunctional thoughts and behaviors. This comprehension may facilitate the development of a more adaptive psychological functioning, which in turn would lead to the improvement of the symptomatic expression.

Moreover, this is the first study exploring the association between ON and the entire psychopathological spectrum, to better understand the differences and the overlapping aspects between ON and other psychopathological conditions. Our results propose that orthorexic individuals are characterized by similar clinical features and defensive functioning as those observed in traditional EDs, suggesting the importance of deepening our understanding of the relationship between these conditions.

## What is already known on this subject?

Previous studies have shown that ON is associated with other psychopathological conditions, particularly EDs and OCD. However, the nature of these relationships needs to be further explored.

## What your study adds?

This study expands the comprehension of ON by exploring the defensive functioning of these individuals, as well as the relationship between this condition and the entire psychopathological spectrum.

## Data Availability

The data that support the findings of this study are available upon reasonable request from the corresponding author.
